# Supplementary material for: The Role of Aspergillus niger in Regulating Internal Browning Involves Flavonoid Biosynthesis and the Endophytic Fungal Community of Pineapple
Source: J Fungi (Basel). 2024 Nov 15;10(11):794. doi: 10.3390/jof10110794 (PMC11595779; doi:10.3390/jof10110794)
Supplement: Supplementary file 1 [file jof-10-00794-s001.zip › Fig. S1 to 3.pdf]

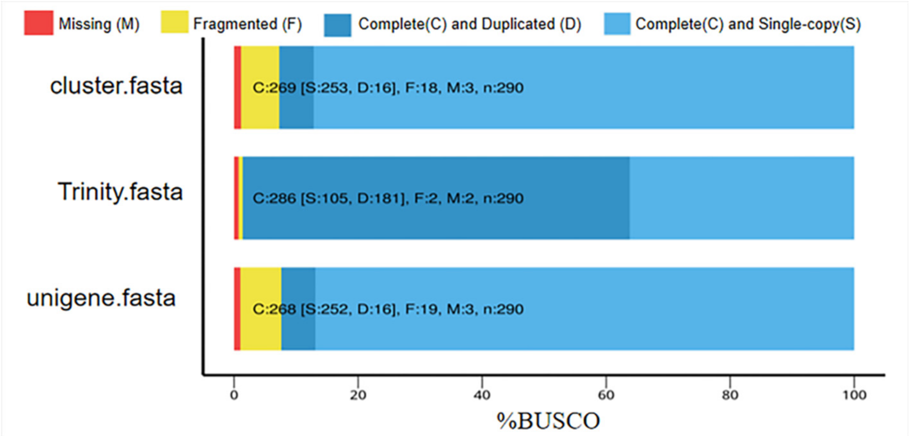

Figure S1 BUSCO evaluation results of spliced transcripts of endophytic fungus *A. niger* and its mutant strain AnM. S, Complete Single-Copy BUSCOs; D, Complete Duplicated BUSCOs; F, Fragmented BUSCOs; M, Missing BUSCOs; n, Total BUSCO groups searched.

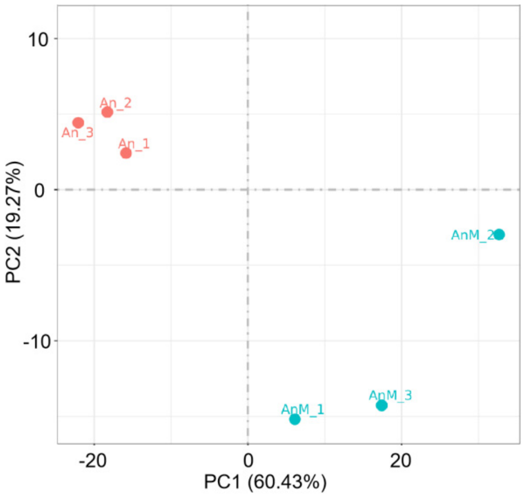

Figure S2 Principal component analysis (PCA) of each transcriptome of endophytic fungus *A. niger* and its mutant strain AnM. An, *A. niger*; AnM, the mutant strain of *A. niger* marked AnM.

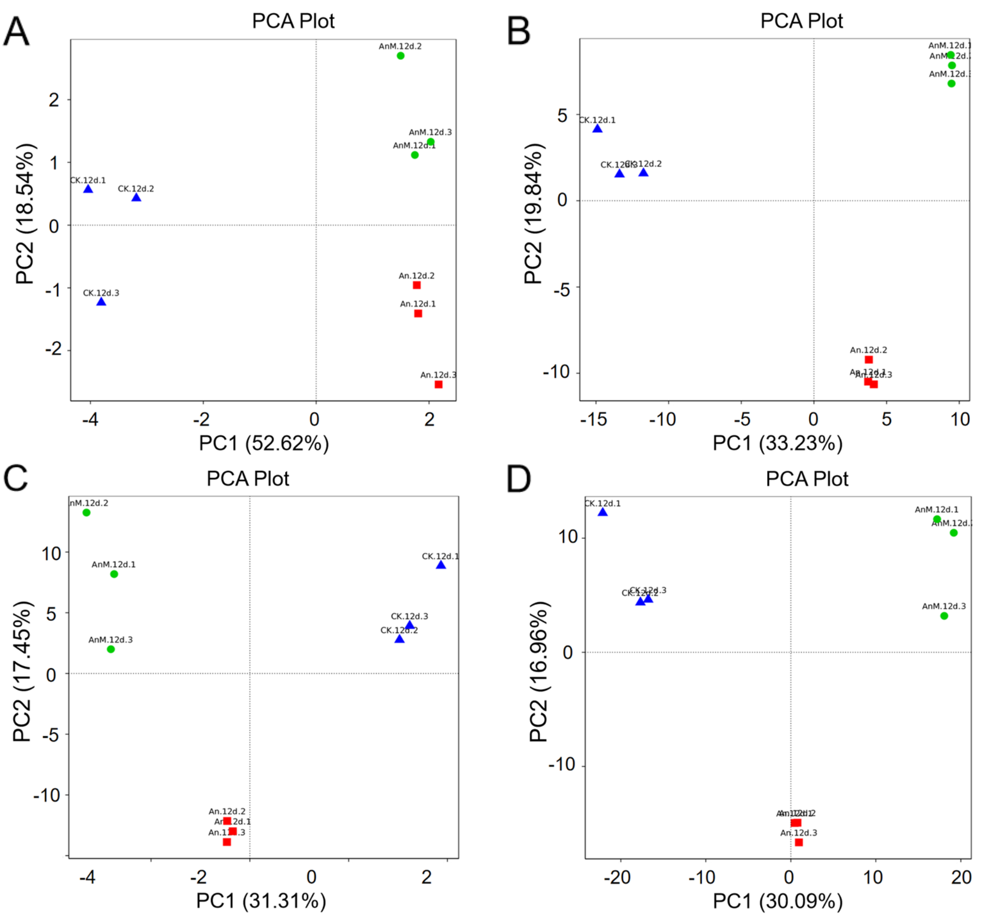

Figure S3 Principal coordinate analyses (PCoA) plots of endophytic fungi in pineapple inoculated by *A. niger* and its mutant strain AnM based on phylum (A), family (B), genus (C), specie (D) level (date from ITS rRNA amplicon sequencing). CK.12d, the control fruit following 12 d storage; An.12d, *A. niger*-inoculated fruit following 12 d storage; AnM.12d, the mutant strain AnM-inoculated fruit following 12 d storage.
